# Adolescents’ Psychological Well-Being: A Multidimensional Measure

**DOI:** 10.3390/ijerph15102325

**Published:** 2018-10-22

**Authors:** Carmen Viejo, Mercedes Gómez-López, Rosario Ortega-Ruiz

**Affiliations:** Department of Psychology, Universidad de Córdoba (Spain), 14004 Córdoba, Spain; cviejo@uco.es (C.V.); ortegaruiz@uco.es (R.O.-R.)

**Keywords:** positive development, well-being, adolescence, questionnaire validation

## Abstract

Over the last few years, different theoretical approaches have emerged advocating for a positive understanding of adolescence, recognizing it as a stage characterized by plasticity, the acquisition of competences and the achievement of satisfactory levels of well-being and positive adjustment. Based on Ryff’s multidimensional model of psychological well-being, this study aims: (1) to develop an adjusted measuring instrument for adolescents (Brief Scale of Psychological Well-Being for Adolescents), as well as analysing its psychometric properties; and (2) to conduct a descriptive analysis of the levels of psychological and subjective well-being among adolescent boys and girls. A sample of 1590 Andalusian adolescents (51% girls), aged between 13 and 19 years old participated in this study. The Confirmatory Factor Analysis (CFA) showed the validity of the instrument, with a multidimensional factorial solution of four factors (self-acceptance, positive interpersonal relationships, autonomy and life development) with good levels of internal consistency. Descriptive analyses showed good scores of psychological and subjective well-being among the adolescents, with a significant impact of sex and age in both measures of well-being. The results are discussed in terms of the importance of considering adolescent well-being from a multidimensional view and the need to promote positive development from a multifactorial perspective which takes into account the diversity of the variables involved.

## 1. Introduction

Psychological research and practice have traditionally focused on studying and treating pathologies following a deficit approach, which has as its main priority to lessen the unpleasant effects of different individual and social problems [1]. This approach has been particularly evident in studies of adolescents, as this period of life involves many changes and has long been portrayed as a time of stress and hardship. This dramatic view of adolescence has been present since Hall put forward in his seminal work the well-known notion of storm and stress [2]. The idea of adolescence as a period in which young people go through emotional and behavioural upheaval [3] was present in subsequent scientific formulations. Anna Freud, for example, pointed out the inevitable problems arising in this life stage and viewed it as a universal period of developmental disturbance [4]. Similarly, Erikson established the identity crisis as a characteristic feature of adolescence [5,6]. In this way, during the first half of the 20th century, most writing and research about adolescence was based on this deficit conception of young people [7], which still lingers on in current social representations [8]. In the same vein, a number of studies have alluded to the increased conflict with parents [9,10], emotional instability [11,12] or risk behaviour [13], which occur during these years and which have led to our image of adolescence as a period of generalized problems. This approach, however, may have had negative consequences for the boys and girls themselves [8]: the lack of empirical and social attention given to the opportunities that this stage in life offers, as well as the contributions that adolescents can make both to the environment and to themselves, may have built up a deep-set negative bias in our understanding of this stage of development.

### 1.1. The Well-Being of Adolescents

To counter the traumatic, conflictive image traditionally associated with adolescence, a stream of studies has emerged in recent years which portray the adolescent not as a source of problems but rather as a valuable asset in a development process [14,15,16]. One example of these alternative approaches is the positive youth development (PYD) perspective. This strength-based view of adolescents [17], points out that the multiple changes that occur during adolescence represent the plasticity inherent in the system of development [18], which represents a fundamental strength of the adolescent period [19], in that it provides the potential for positive functioning [17]. One essential part of this is promoting beneficial relationships between adolescents and their environment, since such adaptive regulations increase the likelihood that healthy and positive changes will take place [17]. Therefore, this perspective encourages us to study adolescents from a broader perspective, such as the development of human potentialities in different areas—physical, psychic and social—which led to a positive view of their general health, rather than focusing on the presence or lack of diseases or disorders [20]. A close empirical perspective to PYD promotion is the one provided by Positive Psychology in relation to the well-being construct. During the last decade, this field has stimulated considerable research aimed at redressing the scientific and practical imbalance in psychopathology between strengths and well-being [21], while paying greater attention to the complementary study of development and the enhancement of human strengths and environmental resources [22]. Nonetheless, most research into human well-being has been carried out on the adult population. During adolescence, well-being seems to have its own developmental idiosyncrasy and differs significantly from later ages [23]. The manifold changes that occur at these ages (physical, physiological, cognitive, emotional, behavioural, social, relational and institutional), make adolescence an exceptionally *plastic* period of life [24], in which the interaction with family, peers and the community should be considered as essential aspects of positive development, capable of generating well-being [25] and as good indicators of positive adjustment at these ages. A society which has healthy, dynamic and cooperative young people is essential for our future economic and social well-being [26] and so identifying and investing in the factors which contribute to the promotion of well-being at pre-adult ages continues to be an important area of research [27].

### 1.2. The Need for Validated Research Tools

In operative terms, the conceptual delimitation of this construct is complex, which is why most studies argue in favour of recognizing two perspectives: the hedonic and the eudaimonic [28]. In the first case, the predominant concept is subjective well-being [29], understood in terms of the balance between positive and negative affect and satisfaction with life. Subjective well-being is therefore made up of both an affective and a cognitive component [30]. The eudaimonic angle, on the other hand, puts the focus on psychological well-being and considers the deployment of skills and personal growth as the main indicators of positive functioning [31]. Although, originally, these two perspectives were conceived as independent [32], the most recent works tend to consider that the measurements carried out under the hedonic and eudaimonic concepts result in different but interrelated constructs [30]. As a result, a blend of both perspectives seems to be the most suitable way to evaluate how people function best, whatever the age but in particular during adolescence [32].

Currently, the topic of subjective well-being has received the most empirical support and has been analysed in different stages of life with research tools validated in different languages [33,34,35,36,37,38]. Self-report questionnaires are among the most commonly used measurements to evaluate positive and negative emotions [39], such as the Positive and Negative Affect Schedule (PANAS) [40], or the Multiple Affect Adjective Checklist-R (MAAC-R) [41]. Subjective well-being has also been measured by evaluating satisfaction with life, with instruments such as the Satisfaction With Life Scale (SWLS) [42] and the Personal Wellbeing Index (PWI) [43]. All these instruments have been widely used in the study of adolescent well-being [44,45,46,47,48,49,50]; however, very few studies address the measurement of psychological well-being in adolescence. Consequently, few research tools are backed up by solid empirical support to measure this construct at this stage of development. The increasing interest in this area of research therefore calls for valid and reliable instruments that measure the essential constructs of positive functioning [51].

### 1.3. The BSPWB-A Questionnaire Development: Theoretical Principles

Ryff’s [52,53] theoretical contributions regarding psychological well-being have laid the foundations for developing one of the most commonly used instruments for measuring it [54,55]. On the basis of perspectives such as Maslow’s conception of self-actualization [56], Rogers’s view of the fully functioning person [57], Allport’s conception of maturity [58] or Erikson’s psychosocial stage model [5] (for review, see [52,59]), Ryff proposed six dimensions to put into practice and measure the eudaimonic approach to the concept of well-being [60]: (1) autonomy, understood by the capacity to have the strength to follow personal convictions, even if they go against conventional wisdom; (2) environmental mastery, which includes being able to manage the demands of everyday life; (3) personal growth, understood by feeling that personal talents and potential are being fulfilled over time; (4) positive relationships with others, in terms of close, valued significant connections with others; (5) purpose in life, in other words, to have goals and objectives that give life meaning and direction; and (6) self-acceptance, or the capacity to see and accept one’s strengths and weaknesses (see [52,53] for complete descriptions). Based on these categories, Ryff [52] developed the Scales of Psychological Well-Being (SPWB), a 120-item research tool targeted at the adult population. Given the difficulties involved in using such an extensive questionnaire, later versions with 84, 54 and 18 items were developed [61,62].

Despite the fact that this research tool has been widely used [63,64,65,66,67], van Dierendonck [51] concluded that to achieve good internal consistency, the scales had to be longer than in the 18-item version but factorial validity required that they be shorter than in the 84- and 54-item versions. Therefore, van Dierendonck presented a new 39-item version [51], whose psychometric properties were improved with respect to previous versions, while keeping the theoretical model of six dimensions proposed by Ryff [52]. Following this version, Díaz et al. [31] developed a Spanish adaptation of this scale for an adult population, producing a shorter, 29-item version validated in Spanish. Despite having fewer items, the scales showed an internal consistency similar to that seen in the scales proposed by van Dierendonck [51] and kept the 6 original dimensions proposed by Ryff [52]. These scales, either in the 39-item or 29-item version, have been used with adolescent samples [68,69,70] but there are still very few studies which analyse the psychological well-being in this specific stage of life. On the one hand, there is a single, short 13-item scale (the Psychological Well-Being Scale, known as ‘BIEPS’ in Spanish) [71,72] to evaluate psychological well-being during adolescence, although there is limited empirical evidence for its reliability, validity and adjustment to the original theoretical model proposed by Ryff [52]. On the other, another adaptation for adolescents of Ryff’s scales was proposed in the study carried out by Loera-Malvaez et al. [70]. These authors produced a 4-factor solution using the 39-item version which coincides partly with the categories designed by Ryff [52,53,60]: self-acceptance, interpersonal relationships, autonomy and satisfaction with life. These authors point out that, at these ages, the differentiation between aspects such as control over one’s environment or having a purpose in life cannot be measured properly, as boys and girls of this age group find it hard to differentiate between these categories. Other authors point also to the high correlations between these scales and those of *self-acceptance* and *personal growth*, which suggests the existence of a degree of overlap and lack of empirical differentiation between them [51,52,61,63,73].

### 1.4. The Present Study

Although the study of eudaimonic well-being has been given a strong empirical impulse in recent years [31,52,53,61,74,75,76,77] there are still gaps in our knowledge which need to be filled. Currently, as far as we know, there is no short research tool, translated into Spanish and validated with adolescents, which enables us to carry out studies linking Psychological Well-being with other personal and contextual variables in boys and girls of these ages. For this reason, the purpose of this work was to develop an instrument for measuring psychological well-being which would meet three basic requirements: (1) it should be in Spanish; (2) it should be adapted to the adolescent population; (3) it should be a short validated version which can be used together with other measuring instruments.

Therefore, the objective of this work is twofold: (1) to design a short research tool for measuring well-being which is in Spanish and is adjusted for adolescents (Brief Psychological Well-Being Scale for Adolescents, BSPWB-A) and analyse its psychometric properties; and (2) to carry out a descriptive analysis of both psychological and subjective well-being in a sample of Spanish adolescents.

## 2. Materials and Methods

### 2.1. Participants

This study was carried out in two stages (pilot scheme and final implementation). A total of 1590 adolescents (49% boys, 51% girls) took part in the final implementation stage, all of whom attended school in state and private schools in the Autonomous Community of Andalusia (Spain) and were aged between 13 and 19 years old (mean age = 15.34; SD = 1.24).

### 2.2. Measures

The research tool we designed was made up of the following scales:

Socio-metric data: a series of open questions which collected information on the personal variables of the participants: sex, age, type of school, school ownership, course and location.

Scale of subjective well-being: the Personal Wellbeing Index (PWI-7) [78] was used, in its adapted version in Spanish for adolescent populations [34]. The scale consists of seven items assessed on a 10-point Likert scale ranging from no satisfaction at all to completely satisfied, which measure adolescents’ satisfaction with different areas of their lives: health, standard of living, achievements in life, personal safety, groups of belonging, security for the future and interpersonal relationships. The average scores derived from the PWI-7 constitute a measure of Subjective Well-Being (*α* = 0.88). In order to calculate and interpret the global index of subjective well-being, the scores are standardized; the normal range for Western populations is between 70 and 80 points [43].

Scale of psychological well-being: BSPWB-A. The adaptation of this instrument was the first stage of this study. Using the version by van Dierendonck [51] as a starting point, the following two adaptations in Spanish have been used: (1) the short 29-item version aimed at the adult population developed by Díaz et al. [31] and (2) the longer, 34-item version validated with an adolescent population developed by Loera-Malvaez et al. [70]. We worked on both questionnaires and all the necessary adaptations and combinations were carried out before ending up with a final version which met all the established requirements (short version, in Spanish, for adolescents). A preliminary 21-item version of the BSPWB-A was obtained and subjected to a piloting process to assess its suitability in terms of linguistic, semantic and functional validity [79]. In this pilot study, 197 adolescents (51% boys, 49% girls) participated, all of whom were enrolled in state schools in the province of Córdoba (Andalusia, Spain) and aged between 12 and 18 years old (mean age = 15.14; SD = 2.48). This version kept the 4 scales which have been identified as necessary for measuring psychological well-being by Loera-Malvaez et al. [70], although by making a few adjustments to the theory and the content, some of the categories were renamed. The final version of the instrument was taken from the pilot study and included a total of 20 items, after one item, which negatively affected the questionnaire’s internal consistency, was deleted. Six more items were reformulated after finding that the participants did not understand what they meant. Finally, taking the model established by Loera-Malvaez et al. [70] as a reference, we decided to validate a 4-factor model which allowed us to identify levels of well-being. The final scale was entitled the Brief Scale of Psychological Well-Being for Adolescents (BSPWB-A), which is an adaptation of the Psychological Well-Being Scales developed by Ryff [52]. The final questionnaire and its relationship with the scales which were used as reference values can be found in Appendix A. The scale is made up of 20 items assessed on a 6-point Likert scale ranging from completely disagree to completely agree, which measure the degree of agreement or disagreement with different statements about 4 areas: self-acceptance, positive interpersonal relationships, autonomy and life development.

### 2.3. Procedure

The research design was cross-sectional, ex-post facto and retrospective, with a single group and multiple measurements [80]. Before the data was collected, the participants’ families gave their informed consent and permission was given by the school. The students answered the questions during school hours in groups: they had previously been informed that their participation would be anonymous, confidential and voluntary. The average time taken to complete the questionnaire was 20 min. The data collection process was carried out by research assistants, who did not belong to the schools. The study was carried out in accordance with the Declaration of Helsinki. Ethics approval was obtained from the Research Ethics Committee of the University of Cordoba, Spain.

### 2.4. Data Analysis

The descriptive and correlational analyses were carried out using the SPSS 20.0 statistical software program (IBM, Armonk, NY, USA), while EQS 6.2 was used for the factorial validity analyses (Multivariate Software, Inc., Encino, CA, USA).

For the Confirmatory Factorial Analysis (CFA), the works of Diaz et al. [31] and Loera-Malvaez et al. [70] were used as a theoretical framework and the suitability of their models was tested with the BSPWB-A. As the instrument was measured on a 6-point Likert scale, we decided to apply the corresponding polychoric correlations [81]. The Maximum Likelihood (ML) estimation method was used, considering that, when used with structural equation models, it was shown to be fairly robust in situations where basic assumptions such as multivariate normality are violated [82]. However, with a Mardia coefficient which indicated an abnormal multivariate distribution, the data analysis was performed with robust coefficients. To evaluate the model, the Satorra-Bentler parameter values were used, corrected according to a robust covariance matrix, as these were seen to provide the most reliable adjustment statistics [83]. In addition to the Satorra-Bentler Chi-Square value, the adjustment values of these models suit the values of the comparative fit index (CFI), the Bentler-Bonett non-normality fit index (NNFI) (≥0.90 suitable, ≥0.95 optimum) and the root mean square error of approximation (RMSEA) (≤0.08 adequate, ≤0.05 optimal) [84]. Finally, we estimated the internal validity of each factor, in addition to the total scale with the ordinal Alpha [85], using a minimum cut-off point of 0.70 [86]. To evaluate convergent validity, the standardized factorial weights were examined, following the criterion of Worthington & Whittaker [87] (values > 0.40 indicate a good convergent validity).

Descriptive analyses on well-being were performed. Comparison analyses with age and gender were also carried out using ANOVA and Student’s t-test, since these two tests are both robust in cases of non-compliance with normality [88]. The effect size was controlled using the Cohen’s d and Eta-Squared statistics.

## 3. Results

### 3.1. Validation of the Instrument: Brief Scale of Psychological Well-Being for Adolescents (BSPWB-A).

The final 20-item version of the BSPWB-A was validated, after carrying out a CFA. A Mardia coefficient score of 115.6968 showed that the data did not meet the assumption of multivariate normality. Inter-item polychoric correlation analyses showed low-mid values, which was indicative of low collinearity (see Table 1).

The CFA showed that the model was correctly adjusted: *χ*^2^S−B = 1487.7853; df = 164; *p* < 0.001; NNFI = 0.925; CFI = 0.935; RMSEA = 0.072 (90% CI [0.069, 0.075]). The internal reliability of each scale and of the questionnaire as a whole, measured with the Alpha Ordinal, had values of over 0.7 in all cases (*α*_self-acceptance_ = 0.87; *α*_positive interpersonal relationships_ = 0.79; *α*_autonomy_ = 0.80; *α*_life development_ = 0.83; *α*t_otal well-being_ = 0.95).

All the items showed high values in terms of standardized factorial weight, which was over 0.49 and significant in all cases, which indicated a good convergent validity (see Table 2). The low but significant covariances between the factors indicated related but not overlapping dimensions.

### 3.2. Psychological and Subjective Well-being in Adolescence by Age and Gender

The second main objective was to make progress in the analysis of the levels of psychological and subjective well-being found in adolescent boys and girls, taking into account any possible differences depending on sex or age. The data produced medium-high scores on all the psychological or subjective welfare scales considered.

The analysis of the influence of gender showed significant differences in the scales of subjective well-being (see Table 3) referring to satisfaction with one’s standard of living (*t* (1560) = 3.21; *p* = 0.001; *r* = −0.08), satisfaction with personal safety (*t* (1543.65) = 7.03; *p* = 0.000; *r* = −0.17) and satisfaction with security for the future (*t* (1559) = 2.64; *p* = 0.008; *r* = −0.07) and in the scales of psychological well-being referring to self-acceptance (*t* (1518.09) = 6.49; p = 0.000; *r* = −0.16) and life development (*t* (1470.37) = −4.57; *p* = 0.000; *r* = −0.12). In all cases, the boys scored higher, except on the scale of development in life, where the girls had higher scores.

Age also proved to have a significant impact on adolescent well-being, with differences in the subjective well-being scales of satisfaction with one’s standard of living (*F* (1559,1564) = 2.35; *p* = 0.039, *η*^2^ = 0.007), satisfaction with one’s achievements in life (*F* (1557, 1562) = 4.88; *p* = 0.000, *η*^2^ = 0.02), satisfaction with personal safety (*F* (1555.1560) = 4.99; *p* = 0.000, *η*^2^ = 0.02) and satisfaction with security for the future (*F* (1558, 1563) = 25.49; *p* = 0.000, *η*^2^ = 0.08) and in the psychological well-being scales of self-acceptance (*F* (1520, 1525) = 4.64; *p* = 0.000, *η*^2^ = 0.02) and life development (*F* (1518, 1523) = 8.93; *p* = 0.000, *η*^2^ = 0.03). In all cases, there were clear differences between the younger groups (13, 14 and 15 years old) and the older groups, with the latter obtaining the lowest scores (see Table 4), except for the *life development* scale, in which older adolescents obtained the highest scores.

## 4. Discussion

Scientific interest in the positive aspects of human functioning has increased considerably in recent decades, giving rise to new definitions, models and measurements of a concept that is still rather ambiguous but is currently understood by well-being [89,90,91]. There has been a marked increase in studies which call for a new perspective on the psycho-evolutionary and change process of adolescence, which highlights the human potential and the values of growth and a positive outlook on life, even in a period which has nearly always previously been looked on as problematic [9,10,11]. This positive approach to adolescence raises the notion that even difficulties and challenges are factors which could contribute to the pursuit of happiness and lead to positive changes [14,15,16,17,19]. However, the study of personal and social life during adolescence has been traditionally plagued with an imbalance towards identifying pathologies and damage-repair, following a model of pathology and conflicts and neglecting any potential for trying to induce young people to be well integrated in their communities or well oriented towards the pursuit of happiness [92]. Due to this, these studies still have difficulties to overcome: from the difficulty of defining key constructs such as well-being, to the lack of suitable research tools which could enable us to obtain valid and reliable scientific evidence from these constructs.

### 4.1. The BSPWB-A Validation

With the aim of contributing to the progress in this field of study, the main objective of this work was to design and validate a brief research tool in Spanish to measure psychological well-being in the adolescent population, as well as analysing its psychometric properties. To do this, we based our study on the adaptations made by Díaz et al. [31] and Loera-Malvaez et al. [70] of the Psychological Well-being Scales developed by Ryff [52]. The results obtained support the BSPWB-A as a valid and reliable instrument, with good indexes of internal consistency and a factorial adjustment with a 4-factor model based on the scales of self-acceptance, positive relationships, autonomy and personal growth proposed by Ryff [52]. Because of the high correlations shown in previous studies [51,52,61,63,73], the scales of *environmental mastery* and *purpose in life* proposed by Ryff [52] were not included. Loera-Malvaez et al. [70] also indicated that these dimensions, due to their content, could be inappropriate in studies with adolescents. The instrument presented in this work showed better internal consistency indices in comparison with both versions used as references, specifically with respect to the autonomy and personal growth (life development in the BSPWB-A) scales [31,70]. In addition, the self-acceptance scale of BSPWB-A had better internal consistency than the version developed by Díaz et al. [31], as did the positive interpersonal relationships scale in comparison with the study carried out by Loera-Malvaez et al. [70]. It is important to highlight that, in these two studies, the personal growth scale showed the lowest alpha coefficients. Our version therefore appears suitable for use with adolescent samples.

### 4.2. Adolescent Well-Being: Associations with Gender and Age

Taking into account the studies which suggest that measuring both psychological and subjective well-being together is the most suitable way of evaluating positive functioning [32] and that both processes of well-being can work together [76], we established as the second main objective the analysis of the rates of psychological and subjective well-being reported by boys and girls of these ages: high scores on both scales would indicate optimal functioning [32] and a better personal adjustment. In this work, the results showed that the average level of the adolescents’ well-being met the requirements to be considered satisfactory, with subjective well-being scores within the established normative range for the western population and medium-high scores for psychological well-being. Although no studies were found which use a similar joint approach to well-being in adolescence, including both subjective and psychological well-being measurements, prior studies have produced similar results regarding subjective well-being [93,94]. The gender differences observed suggest that boys express a higher level of general well-being than girls, although the girls score higher in the items referring to their own life development and satisfaction with life. Previous studies, however, showed no significant gender differences regarding subjective well-being [95,96]. With regard to psychological well-being, studies with adult samples showed that women consistently rate themselves higher in positive relations with others than men and they tend to score higher than men on personal growth [52,61,97], although no studies were found using adolescent samples. In our study, girls scored significantly higher that boys in the *life development* scale (corresponding to *personal growth* in Ryff’s scale), which could point to a possible developmental trend. Further longitudinal studies should be carried out to verify if there are, indeed, gender differences in well-being across age.

As regards age, there were important differences between the younger age group (representing the first stage of adolescence: 13, 14 and 15 years old) and the older age group (representing the phase of late adolescence or the beginning of adulthood: aged 16, 17, 18 or over). The former showed significantly higher levels of well-being than the latter, which could be explained by the different psycho-evolutionary characteristics between the age groups of young adolescents and early adulthood, such as leaving home, looking for work or making future plans as an adult, which can play an important role in the awareness of the contextual variables which facilitate or hinder the achievement of such objectives and have a direct impact on the feeling of well-being shown. Overcoming the psycho-evolutionary tasks presented in this period of the life cycle, some of which are directly linked to the economic, educational, work and social opportunities offered by their social context, leads to improved levels of well-being and personal satisfaction [98]. The decrease in well-being with age found in this work is consistently with other studies of subjective well-being in adolescence [99]. However, no studies have been found analysing age differences in psychological well-being. On this subject, studies with adult samples have suggested that certain aspects of well-being, such as autonomy and environmental mastery, increase with age, while others decrease, such as personal growth and purpose in life [52,61,97,100]. Nonetheless, our results showed an increase in personal growth with age (or life development).

These findings suggest, on the one hand, that adolescence may not be the problematic and turbulent stage of life that still features so prominently in current social representations and research [7,8], lending therefore empirical support to current theories and models of positive development and well-being during adolescence. On the other hand, they promote the idea of adolescence as a period of life in which valuable resources are created [14,15,16] and emphasizing the importance of the existence of beneficial, mutual and healthy links with the environment in which adolescents grow up.

### 4.3. Limitations and Future Directions

Despite all this, it is important to note the limitations of this study. The use of self-report questionnaires on aspects related to happiness or well-being could be affected by what some authors refer to as a *positivist bias*, or a tendency to overestimate the real values of well-being [101,102]. Although this bias has proved to be cross-cultural, future studies using the BSPWB-A in other populations than the Spanish one could help us, on the one hand, to understand how marked this effect is in the study population and on the other, to prove its psychometric properties and invariance to other samples, thus contributing to the generalization of the results. The cross-sectional nature of this work may represent another limitation. Thus, it would also be interesting to develop longitudinal studies which enable us to assess the role of well-being as a cause or consequence of some of the most widely used contextual variables linked to risk and deficit. Finally, future studies including both subjective and psychological well-being measurements will be needed to advance our scientific knowledge about the positive aspects of adolescent development. According to the literature, both types of well-being can operate in tandem, providing evidence that the best psychological outcomes can be obtained through the synergy of both hedonic and eudaimonic processes [32,76,103]. The use of distinct labels and terms therefore leads to thinking about constructs that are separated from one another, which may represent a major obstacle in the progress towards the understanding of positive aspects of human functioning.

## 5. Conclusions

This study supports the idea of considering adolescence as a stage of opportunities and positive development, which in turn allows contemporary systemic models of development to be adopted, focusing on the study of the links between the individual and their context [24]. The focus on the organism-context as a unit of analysis [104] works on the principle that the process of human development involves mutually influential relationships with the environment [105,106] which, when mutually beneficial, become adaptive regulations for development [105,107,108,109]. Although relationships with our environment occur throughout our lives [110,111], they are of special importance in the study of well-being and positive functioning during adolescence, given the plasticity which characterizes this stage of human development [24]. The promotion of well-being during adolescence can help to obtain positive results, as well as acting as a buffer against negative results, such as psychological disorders [112]. In this way, well-being not only represents a key indicator of positive development, but it can also serve to ensure optimal mental health [113] and to discover the beneficial adjustment pathways between adolescents and their context, leading to greater chances of achieving positive changes in the transition to adulthood [114,115,116].

## Figures and Tables

**Table 1:** Polichoric correlation matrix: BSPWB-A (Brief Psychological Well-Being Scale for Adolescents).

Items	PW1	PW2	PW3	PW4	PW5	PW6i	PW7i	PW8	PW9i	PW10	PW11i	PW12i	PW13i	PW14i	PW15i	PW16i	PW17	PW18	PW19	PW20
PW1	1	-	-	-	-	-	-	-	-	-	-	-	-	-	-	-	-	-	-	-
PW2	0.62	1	-	-	-	-	-	-	-	-	-	-	-	-	-	-	-	-	-	-
PW3	0.47	0.67	1	-	-	-	-	-	-	-	-	-	-	-	-	-	-	-	-	-
PW4	0.61	0.69	0.71	1	-	-	-	-	-	-	-	-	-	-	-	-	-	-	-	-
PW5	0.35	0.47	0.52	0.54	1	-	-	-	-	-	-	-	-	-	-	-	-	-	-	-
PW6i	0.19	0.24	0.24	0.27	0.09	1	-	-	-	-	-	-	-	-	-	-	-	-	-	-
PW7i	0.24	0.26	0.23	0.29	0.11	0.73	1	-	-	-	-	-	-	-	-	-	-	-	-	-
PW8	0.28	0.29	0.30	0.36	0.27	0.37	0.35	1	-	-	-	-	-	-	-	-	-	-	-	-
PW9i	0.15	0.19	0.17	0.19	0.13	0.52	0.54	0.29	1	-	-	-	-	-	-	-	-	-	-	-
PW10	0.30	0.30	0.32	0.35	0.30	0.37	0.38	0.65	0.33	1	-	-	-	-	-	-	-	-	-	-
PW11i	0.12	0.19	0.19	0.19	0.17	0.21	0.24	0.05	0.20	0.02	1	-	-	-	-	-	-	-	-	-
PW12i	0.14	0.25	0.24	0.24	0.14	0.24	0.26	0.01	0.14	0.05	0.66	1	-	-	-	-	-	-	-	-
PW13i	0.29	0.36	0.35	0.36	0.19	0.29	0.28	0.08	0.19	0.13	0.37	0.45	1	-	-	-	-	-	-	-
PW14i	0.24	0.28	0.25	0.29	0.13	0.30	0.36	0.14	0.24	0.13	0.29	0.35	0.43	1	-	-	-	-	-	-
PW15i	0.42	0.40	0.37	0.47	0.25	0.35	0.41	0.26	0.35	0.26	0.36	0.33	0.49	0.54	1	-	-	-	-	-
PW16i	0.09	0.13	0.17	0.15	0.20	0.22	0.28	0.09	0.26	0.10	0.35	0.38	0.37	0.39	0.40	1	-	-	-	-
PW17	0.20	0.23	0.24	0.26	0.29	0.11	0.13	0.35	0.10	0.36	0.03	0.02	−0.02	0.06	0.12	−0.002	1	-	-	-
PW18	0.26	0.27	0.26	0.29	0.27	0.09	0.16	0.41	0.09	0.34	0.006	−0.02	−0.001	0.09	0.24	0.05	0.68	1	-	-
PW19	0.15	0.17	0.18	0.21	0.22	−0.002	0.06	0.29	0.08	0.25	−0.02	−0.01	−0.06	0.04	0.14	0.002	0.47	0.67	1	-
PW20	0.28	0.35	0.31	0.32	0.30	0.14	0.18	0.33	0.13	0.28	0.02	0.04	0.08	0.12	0.23	0.05	0.45	0.58	0.48	1

Note: PW: Psychological Well-being; i = item with inverted scores.

**Table 2:** Standardized solutions for CFA (Confirmatory Factor Analysis) of the BSPWB-A (Brief Psychological Well-Being Scale for Adolescents).

		Standardized Solution	R-Square	Alpha Ordinal
factor 1	PW1	0.682 f1 + 0.731 e1	0.465	0.87
	PW2	0.819 * f1 + 0.573 e2	0.671	
	PW3	0.803 * f1 + 0.596 e3	0.645	
	PW4	0.874 * f1 + 0.487 e4	0.763	
	PW5	0.603 * f1 + 0.797 e5	0.364	
covariance f1-f2		0.228 *		
covariance f1-f3		0.215 *		
covariance f1-f4		0.193 *		
factor 2	PW6i	0.824 f2 + 0.567 e6	0.679	0.79
	PW7i	0.849 * f2 + 0.529 e7	0.720	
	PW8	0.493 * f2 + 0.870 e8	0.243	
	PW9i	0.631 * f2 + 0.776 e9	0.398	
	PW10	0.515 * f2 + 0.857 e10	0.265	
covariance f2-f3		0.259 *		
covariance f2-f4		0.146 *		
factor 3	PW11i	0.593 f3 + 0.806 e11	0.351	0.80
	PW12i	0.631 * f3 + 0.776 e12	0.398	
	PW13i	0.680 * f3 + 0.733 e13	0.462	
	PW14i	0.641 * f3 + 0.767 e14	0.411	
	PW15i	0.720 * f3 + 0.694 e15	0.519	
	PW16i	0.557 * f3 + 0.830 e16	0.311	
covariance f3-f4		0.051 *		
factor 4	PW17	0.724 f4 + 0.690 e17	0.524	0.83
	PW18	0.927 * f4 + 0.374 e18	0.860	
	PW19	0.710 * f4 + 0.704 e19	0.504	
	PW20	0.636 * f4 + 0.772 e20	0.405	

f = factor; e = error; * *p* < 0.05.

**Table 3:** Mean scores (Standard Deviation) of Subjective Well-being and Psychological Well-being during adolescence (by sex).

Well-Being Scales		Total M (SD)	Boys M (SD)	Girls M (SD)
Subjective well-being	Satisfaction with health	82.34 (18.93)	82.93 (18.81)	81.77 (19.06)
	Satisfaction with one’s standard of living *	82.54 (17.89)	84.04 (17.69)	81.14 (18.01)
	Satisfaction with one’s achievements in life	78.24 (19.55)	78.25 (19.68)	78.29 (19.31)
	Satisfaction with personal safety *	73.39 (23.53)	77.59 (21.35)	69.37 (24.78)
	Satisfaction with groups of belonging	84.90 (18.39)	85.58 (18.05)	84.22 (18.71)
	Satisfaction with security for the future *	74 (22.27)	75.53 (21.54)	72.57 (22.86)
	Satisfaction with interpersonal relationships	81.27 (19.22)	82.05 (19.73)	80.54 (18.73)
Psychological well-being	Self-acceptance *	4.71 (0.86)	4.86 (0.81)	4.57 (0.89)
	Positive interpersonal relationships	4.73 (0.98)	4.74 (0.96)	4.74 (1.00)
	Autonomy	4 (1.02)	4.03 (1.04)	3.98 (1.01)
	Life development *	4.98 (0.86)	4.88 (0.91)	5.08 (0.80)

Note: the Subjective Well-being scores were standardized; according to the authors, the normative range for occidental people is between 70–80 points [43]. The Psychological Well-being scores ranged from 1 to 6. * *p* < 0.05.

**Table 4:** Significant differences on Subjective Well-being and Psychological Well-being by age.

	13 Mean (SD)	14 Mean (SD)	15 Mean (SD)	16 Mean (SD)	17 Mean (SD)	18+ Mean (SD)	*F*	*gl*	*p*	*η* ^2^	(I) Age	(J) Age	Mean Differences (I-J)
Subjective Well-being													
Satisfaction with health	80.52 (20.18)	82.89 (19.69)	82.29 (19.76)	82.35 (18.66)	82.50 (16.53)	80.73 (17.95)	0.29	1559, 1564	0.916	0.001	No post-hoc differences		
Satisfaction with one’s standard of living *	84.17 (16.66)	84.25 (16.34)	82.71 (19.83)	82.35 (18.20)	80.79 (16.50)	77.43 (16.68)	2.35	1559, 1564	0.039	0.007	No post-hoc differences		
Satisfaction with one’s achievements in life *	82.50 (17.46)	80.71 (19.99)	78.72 (21.51)	77.22 (17.61)	75.98 (17.72)	70.49 (20.21)	4.88	1557, 1562	<0.001	0.015	13	18+	12.01 *
											14	17	4.73 *
												18+	10.22 *
											15	18+	8.23 *
Satisfaction with personal safety *	72.60 (25.60)	76.59 (22.46)	74.95 (24.96)	72.96 (21.55)	68.33 (23.55)	67.36 (24.87)	4.99	1555, 1560	<0.001	0.016	14	17	8.26 *
											15	17	6.61 *
Satisfaction with groups of belonging	84.78 (19.2)	86.22 (17.45)	85.11 (19.56)	84.33 (19.11)	83.67 (16.96)	84.03 (15.69)	0.72	1555, 1560	0.608	0.002	No post-hoc differences		
Satisfaction with security for the future *	83.10 (16.68)	79.49 (20.82)	78.01 (22.69)	70.07 (20.67)	65.36 (22.35)	61.46 (22.13)	25.49	1558, 1563	<0.001	0.076	13	16	13.03 *
												17	17.74 *
												18+	21.64 *
											14	16	9.42 *
												17	14.12 *
												18+	18.03 *
											15	16	7.94 *
												17	12.65 *
												18+	16.55 *
Satisfaction with interpersonal relationships	82.19 (20.09)	82.63 (19.04)	82.33 (20.17)	79.83 (19.71)	80.11 (16.63)	78.29 (18.72)	1.58	1557, 1562	0.162	0.005	No post-hoc differences		
Psychological Well-being													
Self-acceptance *	4.84 (0.88)	4.86 (0.84)	4.72 (0.91)	4.66 (0.83)	4.54 (0.82)	4.65 (0.83)	4.64	1520, 1525	<0.001	0.015	14	17	0.31 *
Positive interpersonal relationships	4.60 (1.03)	4.74 (0.98)	4.66 (0.93)	4.77 (1.02)	4.80 (0.99)	4.87 (0.99)	1.27	1519, 1524	0.273	0.004	No post-hoc differences		
Autonomy	4.09 (1.09)	4.12 (1.04)	3.97 (1.05)	3.98 (0.99)	3.89 (0.99)	4.01 (0.91)	1.75	1519, 1524	0.119	0.006	No post-hoc differences		
Life development *	4.80 (0.96)	4.87 (0.96)	4.86 (0.89)	5.08 (0.78)	5.19 (0.68)	5.27 (0.81)	8.93	1518, 1523	<0.001	0.029	14	16	−0.21 *
												17	−0.31 *
												18+	−0.39 *
											15	16	−0.22 *
												17	−0.33 *
												18+	−0.41 *

* *p* < 0.05.

## Appendix A. Reference Scales and Final Questionnaire: Brief Scale of Psychological Well-Being for Adolescents (BSPWB-A)

**Table:**

Scale *	29 Items Version for Adults [31]	34 Items version for Adolescents [70]	BSPWB-A: 20 Items Version for Adolescents	Item Description [53]
Autoaceptación // Self-acceptance	1. Cuando repaso la historia de mi vida estoy contento con cómo han resultado las cosas	1. Cuando repaso la historia de mi vida estoy contento con cómo han resultado las cosas	1. Cuando repaso la historia de mi vida estoy contento con cómo han resultado las cosas // When I look at the story of my life, I am pleased with how things have turned out	To have positive feelings about past life
		6. Disfruto haciendo planes para el futuro y trabajar para hacerlos realidad		To have goals and objectives in life
	7. En general, me siento seguro y positivo conmigo mismo	7. En general, me siento seguro y positivo conmigo mismo	2. En general, me siento seguro y positivo conmigo mismo // In general, I feel confident and positive about myself	To have a positive attitude toward the self
		11. He sido capaz de construir un hogar y un modo de vida a mi gusto		To see improvement in life progress
		12. Soy una persona activa al realizar los proyectos que propuse para mí mismo		To have goals and objectives in life
		16. En general, siento que soy responsable de la situación en la que vivo		To feel competent in managing the environment
		17. Me siento bien cuando pienso en lo que he hecho en el pasado y lo que espero hacer en el futuro		To feel there is meaning to present and past life
		18. Mis objetivos en la vida han sido más una fuente de satisfacción que de frustración para mí		To have goals and objectives in life
	19. Me gusta la mayor parte de los aspectos de mi personalidad	19. Me gusta la mayor parte de los aspectos de mi personalidad	3. Me gusta la mayor parte de los aspectos de mi personalidad // I like most aspects of my personality	To have a positive attitude toward the self
		21. Tengo confianza en mis opiniones incluso si son contrarias al consenso general	4. Tengo confianza en mis opiniones incluso si son contrarias al consenso general // I have confidence in my own opinions, even if they are contrary to the general consensus	To be able to resist social pressures and be determining and independent
		23. Tengo clara la dirección y el objetivo de mi vida		To have goals and objectives in life
		24. En general, con el tiempo siento que sigo aprendiendo más sobre mí mismo		To have a feeling of continuous development
		28. Soy bastante bueno manejando muchas de mis responsabilidades en la vida diaria		To feel competent in managing the environment
		**29. No tengo claro qué es lo que intento conseguir en la vida**		To have lack a sense of direction of own life
	31. En su mayor parte, me siento orgulloso de quien soy y la vida que llevo	31. En su mayor parte, me siento orgulloso de quien soy y la vida que llevo	5. En su mayor parte, me siento orgulloso de quien soy y la vida que llevo // In general, I feel proud of who I am and the life I lead	To evaluate self by personal standards
Relaciones positivas ^1^ // Positive relations with others	**2. A menudo me siento solo porque tengo pocos amigos íntimos con quienes compartir mis preocupaciones**	**2. A menudo me siento solo porque tengo pocos amigos íntimos con quienes compartir mis preocupaciones**	**6. A menudo me siento solo porque tengo pocos amigos íntimos con quienes compartir mis preocupaciones // I often feel lonely because I have few close friends with whom to share my concerns**	To feel isolated and frustrated regarding to interpersonal relationships
		**5. Me resulta difícil dirigir mi vida hacia un camino que me satisfaga**		To have lack a sense of direction of own life
	**8. No tengo muchas personas que quieran escucharme cuando necesito hablar**	8. No tengo muchas personas que quieran escucharme cuando necesito hablar	**7. No tengo muchas personas que quieran escucharme cuando necesito hablar // I don’t have many people who want to listen when I need to talk.**	To have few close, trusting relationships with others
	14. Siento que mis amistades me aportan muchas cosas	14. Siento que mis amistades me aportan muchas cosas	8. Siento que mis amistades me aportan muchas cosas // I feel that my friends bring me a lot of things	To have warm, satisfying and trusting relationships
		**20. Me parece que la mayor parte de las personas tienen más amigos que yo**		To have a feeling of few close, trusting relationships with others
	**26. No he experimentado muchas relaciones cercanas y de confianza**	**26. No he experimentado muchas relaciones cercanas y de confianza**	***9. No he tenido muchas relaciones cercanas y de confianza // I have not experienced many warm and trusting relationships with others***	To have a feeling of few close, trusting relationships with others
	32. Sé que puedo confiar en mis amigos, y ellos saben que pueden confiar en mi	32. Sé que puedo confiar en mis amigos, y ellos saben que pueden confiar en mí	10. Sé que puedo confiar en mis amigos, y ellos saben que pueden confiar en mí // I know that I can trust my friends and they know that they can trust me	To have warm, satisfying and trusting relationships
		37. Tengo la sensación de que con el tiempo me he desarrollado mucho como persona		To perceived self as growing and developing the own potential
Autonomía // Autonomy	3. No tengo miedo de expresar mis opiniones, incluso cuando son opuestas a las opiniones de la mayoría de la gente			To be able to resist social pressures and be determining and independent
	**4. Me preocupa cómo otra gente evalúa las elecciones que he hecho en mi vida**	4. Me preocupa cómo otra gente evalúa las elecciones que he hecho en mi vida	**11. Me preocupa cómo otra gente evalúa las elecciones que he hecho en mi vida. I tend to worry about how other people assess the choices I have made in my life**	To be concerned about the expectations and evaluations of others
	**9. Tiendo a preocuparme sobre lo que otra gente piensa de mí**	9. Tiendo a preocuparme sobre lo que otra gente piensa de mí	**12. Tiendo a preocuparme sobre lo que otra gente piensa de mí // I tend to worry what other people think of me**	To be concerned about the expectations and evaluations of others
		**13. Si tuviera la oportunidad, hay muchas cosas de mí mismo que cambiaría**	**13. Si tuviera la oportunidad, hay muchas cosas de mí mismo que cambiaría // If I had the opportunity, there are many things about myself that I would change**	To feel dissatisfied with the self and wish to be different
	**15. Tiendo a estar influenciado por la gente con fuertes convicciones**			To be able to resist social pressures and be determining and independent
	21. Tengo confianza en mis opiniones incluso si son contrarias al consenso general			To be able to resist social pressures and be determining and independent
		**22. Las demandas de la vida diaria a menudo me deprimen**	***14. Las tareas y obligaciones de mi vida diaria a menudo me deprimen // The tasks and responsibilities of everyday often get me down***	To have difficulty managing everyday tasks and responsibilities
		**25. En muchos aspectos, me siento decepcionado de mis logros en la vida**	**15. En muchos aspectos, me siento decepcionado de mis logros en la vida // In many ways, I feel disappointed about my achievements in life**	To have lack a sense of improvement over time
	**27. Es difícil para mí expresar mis propias opiniones en asuntos polémicos**			To be able to resist social pressures and be determining and independent
		**33. A menudo cambio mis decisiones si mis amigos o mi familia están en desacuerdo**	**16. A menudo cambio mis decisiones si mis amigos o mi familia están en desacuerdo // I often change my mind about decisions if my friends or family disagree**	To rely own decisions in other people’s judgements
Crecimiento personal ^2^ // Personal growth	24. En general, con el tiempo siento que sigo aprendiendo más sobre mí mismo			To have a feeling of continuous development
		**30. Hace mucho tiempo que dejé de intentar hacer grandes mejoras o cambios en mi vida**	(Hace mucho tiempo que dejé de intentar hacer grandes cambios o mejoras en mi vida)	To feel unable to develop new attitudes or make personal progress
		35. Pienso que es importante tener nuevas experiencias que desafíen lo que uno piensa sobre sí mismo y sobre el mundo	*17. Pienso que es importante tener nuevas experiencias que supongan un reto para mí // I think it is important to have new experiences that challenge me*	To be open to new experiences
	**36. Cuando pienso en ello, realmente con los años no he mejorado mucho como persona**	**36. Cuando pienso en ello, realmente con los años no he mejorado mucho como persona**	*18. Creo que todo lo que vivimos son oportunidades para crecer y mejorar como persona // I think everything we experience is an opportunity to grow and to become a better person*	To have lack a sense of improvement over time
	37. Tengo la sensación de que con el tiempo me he desarrollado mucho como persona			To have a feeling of continuous development
	38. Para mí, la vida ha sido un proceso continuo de estudio, cambio y crecimiento	38. Para mí, la vida ha sido un proceso continuo de estudio, cambio y crecimiento	*19. Creo que la vida es un proceso continuo de estudio, cambio y crecimiento // I think life is a continuous process of learning, changing and growth.*	To have a feeling of continuous development
		39. Si me sintiera infeliz con mi situación de vida daría los pasos más eficaces para cambiarla	*20. Cuando encuentro dificultades o no me siento feliz con algo de mi vida, intento buscar un modo de cambiarlo y seguir adelante // When I encounter difficulties, or I do not feel happy with anything in my life, I try to look for a way to change it and move forward*	To change in ways that reflect more effectiveness
Dominio del entorno // Environmental mastery	5. Me resulta difícil dirigir mi vida hacia un camino que me satisfaga	(moved to *positive relationships*)		
	11. He sido capaz de construir un hogar y un modo de vida a mi gusto	(moved to *self-acceptance*)		
	16. En general, siento que soy responsable de la situación en la que vivo	(moved to *self-acceptance*)		
	22. Las demandas de la vida diaria a menudo me deprimen	(moved to *autonomy*)		
	39. Si me sintiera infeliz con mi situación de vida daría los pasos más eficaces para cambiarla	(moved to *personal growth*)		
Propósito en la vida // Purpose in life	6. Disfruto haciendo planes para el futuro y trabajar para hacerlos realidad	(moved to *self-acceptance*)		
	12. Soy una persona activa al realizar los proyectos que propuse para mí mismo	(moved to *self-acceptance*)		
	17. Me siento bien cuando pienso en lo que he hecho en el pasado y lo que espero hacer en el futuro	(moved to *self-acceptance*)		
	18. Mis objetivos en la vida han sido más una fuente de satisfacción que de frustración para mí	(moved to *self-acceptance*)		
	23. Tengo clara la dirección y el objetivo de mi vida	(moved to *self-acceptance*)		

Note: The changed items are in italics; the inversed score items are in **bold**; the item deleted after the pilot study is in between parenthesis. ^1^ In 34 items version for adolescents [70], this factor is named Interpersonal Relationships; in the validated version of BSPWB-A it is called Positive Interpersonal Relationships. ^2^ In 34 items version for adolescents [70], this factor is named Satisfaction with life; in the validated version of BSPWB-A it is called Life development. * Labels of Ryff’s original scales [53].
